# ﻿Multi-gene phylogeny and morphology of two new *Phyllosticta* (Phyllostictaceae, Botryosphaeriales) species from China

**DOI:** 10.3897/mycokeys.95.100414

**Published:** 2023-03-01

**Authors:** Cheng-Bin Wang, Jing Yang, Yong Li, Han Xue, Chun-Gen Piao, Ning Jiang

**Affiliations:** 1 Key Laboratory of Biodiversity Conservation of National Forestry and Grassland Administration, Ecology and Nature Conservation Institute, Chinese Academy of Forestry, Beijing 100091, China Key Laboratory of Biodiversity Conservation of National Forestry and Grassland Administration, Ecology and Nature Conservation Institute, Chinese Academy of Forestry Beijing China; 2 Natural Resources and Planning Bureau of Rizhao City, Rizhao 276827, China Natural Resources and Planning Bureau of Rizhao City Rizhao China

**Keywords:** Ascomycota, morphology, new species, phylogeny, plant disease, taxonomy

## Abstract

*Phyllosticta* (Phyllostictaceae, Botryosphaeriales) includes plant pathogens, endophytes and saprobes, occurring on various hosts worldwide. During the present study, isolates associated with leaf spots were obtained from the hosts *Quercusaliena* and *Viburnumodoratissimum*, and identified based on morphological features and phylogenetic inference from the analyses of five loci (ITS, LSU, *tef1*, *act* and *gapdh*). Results supported the introduction of two novel species, namely *Phyllostictaanhuiensis* and *P.guangdongensis*. Phylogenetically, *P.anhuiensis* and *P.guangdongensis* formed two well-separated lineages in the *P.concentrica* and *P.capitalensis* species complexes, distinguishing from all presently accepted species in this genus by DNA sequence data. Morphologically, *P.anhuiensis* and *P.guangdongensis* have the typical structure of the genus *Phyllosticta*, and differed from their closely related species by the length of the conidial appendage.

## ﻿Introduction

The genus *Phyllosticta* was established by Persoon (1818) and classified in Phyllostictaceae (Botryosphaeriales) (Phillips et al. 2019; Wijayawardene et al. 2020). Initially, *Phyllosticta* was placed in the Phyllostictaceae (Fries 1849). In a multi-locus phylogeny in the Dothideomycetes, Schoch et al. (2006) placed *Phyllosticta* into Botryosphaeriaceae (Botryosphaeriales), which was agreed upon by Crous et al. (2006) and Liu et al. (2012). Subsequently, Slippers et al. (2013) reinstated the Phyllostictaceae to accommodate *Phyllosticta* in terms of phylogenetic relationships. Recently, *Pseudofusicoccum* was added in this family based on the morphological characters of the conidia covered by a mucous sheath and molecular evidence (Phillips et al. 2019). The asexual morph of *Phyllosticta* is characterized by pycnidial conidiomata containing aseptate conidia surrounding with a mucoid layer and bearing a single apical appendage (van der Aa 1973; van der Aa and Vanev 2002; Wikee et al. 2011). The sexual morph of *Phyllosticta* is characterized by erumpent ascomata, 8-spored, clavate to broadly ellipsoid asci, ellipsoid to limoniform ascospores (van der Aa 1973; Wikee et al. 2011). Following the implementation of “one fungus one name” nomenclature rules, the name *Phyllosticta* (asexual state) was used over *Guignardia* (sexual state) and *Leptodothiorella* (spermatial state) (Glienke et al. 2011; Wikee et al. 2011).

The *Phyllosticta* species identification solely delimited by morphology and host association may be difficult to assess (Wikee et al. 2011; Su and Cai 2012). Many species are difficult to distinguish due to slight morphological variation, and the mucoid layer or appendage will be absent or invisible in some species (van der Aa and Vanev 2002; Jin 2011; Wikee et al. 2011). Besides, the host range of *Phyllosticta* is unclear; some species exhibit the broadest host range while others do not (Wikee et al. 2011; Rashmi et al. 2019; Norphanphoun et al. 2020). To overcome the lack of morphological features and host range, phylogenetic approaches based on molecular loci were used to resolve the classification and identification of *Phyllosticta* species (Baayen et al. 2002; Wulandari et al. 2009; Wong et al. 2012; Wikee et al. 2013a). Based on the phylogenetic analyses of a combined ITS, LSU, *tef1*, *act* and *gapdh* sequence data, the current taxonomic classification of *Phyllosticta* comprises six species complexes i.e., *P.capitalensis*, *P.concentrica*, *P.cruenta*, *P.owaniana*, *P.rhodorae* and *P.vaccinii* species complexes (Norphanphoun et al. 2020). Currently, the polyphasic approach involving phylogenetic, morphological, and other analyses is used to clarify species boundaries (Norphanphoun et al. 2020; Zhang et al. 2022).

Members of *Phyllosticta* species are known as pathogenic, endophytic, or rarely saprobic fungi associated with a variety of plants and have a worldwide distribution (van der Aa and Vanev 2002; Glienke et al. 2011; Wikee et al. 2011; Jiang et al. 2021; Wang et al. 2023). As pathogens, *Phyllosticta* species cause spots on the leaves or fruits of many economical plants (e.g., *Musa* spp., *Citrus* spp. and *Vitis* spp.), leading to substantial economic losses (Wang et al. 2012; Wong et al. 2012; Wikee et al. 2013b; Tran et al. 2017). As endophytes, some species were found associated with leaf spots but did not cause any symptom in pathogenicity tests, e.g., *P.oblongifoliae* was isolated from leaf spots of *Garciniaoblongifolia*, *P.pterospermi* was isolated from leaf spots of *Pterospermumheterophyllum*, and *P.capitalensis* was isolated from leaf spots of *Citrus* spp. (Wikee et al. 2013b; Tran et al. 2019; Zhang et al. 2022). In this study, two novel fungal species named *P.anhuiensis* and *P.guangdongensis*, were isolated from diseased leaves of *Quercusaliena* in Anhui Province and *Viburnumodoratissimum* in Guangdong Province, respectively. This paper describes these species based on molecular evidence and morphological characteristics.

## ﻿Materials and methods

### ﻿Isolation and morphological observations

Samples of *Quercusaliena* and *Viburnumodoratissimum* showing necrotic spots were obtained and collected from Anhui and Guangdong Provinces. Samples were surface-sterilized in 75% ethanol for 30 s, then sterilized in 1.5% sodium hypochlorite for 1 min, followed by three rinses with sterilized water and dried on sterilized filter paper, and cut into small sections (3 × 3 mm) from the margins of infected tissues. The sections were plated onto potato dextrose agar (PDA) plates and incubated at 25 °C. Hyphal tips from the edge of emerging colonies were transferred on fresh PDA plates and purified by single-spore culturing (Choi et al. 1999). The cultures and dried specimens of the new isolates have been deposited with the China Forestry Culture Collection Center (**CFCC**; http://cfcc.caf.ac.cn/) and the herbarium of the Chinese Academy of Forestry (**CAF**; http://museum.caf.ac.cn/).

Colony features of cultures on PDA medium, synthetic low-nutrient agar (SNA), and malt extract agar (MEA) were recorded after 14 d incubation at 25 °C. After conidiomata appeared, fungal structures (including conidia, conidiogenous cells, and appendage) were measured and captured at least 50 measurements using a Nikon Eclipse 80i compound microscope with differential interference contrast optics.

### ﻿DNA extraction, PCR amplification, and sequencing

Genomic DNA was extracted from fungal cultures grown on PDA medium using a CTAB method (Doyle and Doyle 1990). Polymerase chain reaction (PCR) amplification of the ITS, LSU, *tef1*, *act*, and *gapdh* loci were amplified using the primers: ITS1/ITS4 (White et al. 1990), EF1-728F/EF2 (O’Donnell et al. 1998; Carbone and Kohn 1999), ACT-512F/ACT-783R (Carbone and Kohn 1999) and Gpd1-LM/Gpd2-LM (Myllys et al. 2002), respectively. Amplification reactions were performed in a 20 μl reaction volume system containing 10 µl of 2× Taq Mix (Tiangen, China), 1 μl of each primer (10 μM), 1 μl template DNA (20 ng/μl) and 7 μL RNase-free water. PCR parameters were as follows: an initial denaturation step of 5 min at 94 °C, followed by 35 cycles of 30 s at 94 °C, 50 s at 55 °C for ITS, 51 °C for LSU, 48 °C for *tef1* or 52 °C for *act* and *gapdh*, and 1 min at 72 °C, and a final elongation step of 10 min at 72 °C. The PCR products were purified and sequenced in Shanghai Invitrogen Biological Technology Company Limited (Beijing, China).

### ﻿Phylogenetic analyses

Newly generated in this study were combined using SeqMan v. 7.1.0, and reference sequences (Table 1) were downloaded from GenBank, according to the recent publication (Hattori et al. 2020; Norphanphoun et al. 2020; Crous et al. 2021; Bhunjun et al. 2022; Nguyen et al. 2022; Tan and Shivas 2022; Zhang et al. 2022). Alignments were done by MAFFT v. 7.036 (https://maft.cbrc.jp/alignment/server/) using default settings and manually improved using MEGA v.7.0 (Kumar et al. 2016). The phylogenetic analyses of the combined five loci (ITS, LSU, *tef1*, *act* and *gapdh*) were performed by maximum likelihood (ML) and Bayesian inference (BI). The ML research was conducted with the CIPRES web portal (Miller et al. 2017) using RAxML v. 8.2.12 (Stamatakis 2014) under the GTR+GAMMA model with 1000 bootstrap iterations. The BI analyses was performed by MrBayes 3.1.2 (Ronquist and Huelsenbeck 2003). MrModelTest v. 2.3 (Nylander 2004) was used to determine the best-fit evolution model for each locus. Bayesian posterior probabilities (BYPP) were evaluated by Markov Chain Monte Carlo sampling (MCMC). Four Markov chains were performed for 2 million generations in two independent runs until the split deviation frequencies decreased below 0.01, and sampling every 100 generations. The first 25% of sampled trees were discarded as burn-in, and the remaining ones were used to calculate BYPP. Trees were visualized in FigTree 1.4 (http://tree.bio.ed.ac.uk/software/figtree), and the ML bootstraps (ML-BS) ≥ 50% and BYPP ≥ 0.9 were presented on nodes of the ML tree.

**Table 1.** Species and GenBank accession numbers of DNA sequences used for phylogenetic analyses in this study.

Species	Strain no.*	Host	Location	GenBank no.				
				ITS	LSU	*tef1*	*act*	*gapdh*
***Phyllostictacapitalensis* species complex**								
*P.acaciigena*	CPC 28295 ^T^	*Acaciasuaveolens*	Australia	KY173433	KY173523	NA	KY173570	NA
*P.aloeicola*	CPC 21020 ^T^	*Aloeferox*	South Africa	KF154280	KF206214	KF289193	KF289311	KF289124
	CPC 21021	*Aloeferox*	South Africa	KF154281	KF206213	KF289194	KF289312	KF289125
*P.ardisiicola*	NBRC 102261 ^T^	*Ardisiacrenata*	Japan	AB454274	NA	NA	AB704216	NA
*P.aristolochiicola*	BRIP 53316 ^T^	*Aristolochiaacuminata*	Australia	JX486129	NA	NA	NA	NA
*P.azevinhi*	MUCC0088	*Ilexpedunculosa*	Japan	AB454302	NA	NA	AB704226	NA
*P.beaumarisii*	CBS 535.87	*Muehlenbekiaadpressa*	Australia	NR_145235	NG_058040	KF766429	KF306232	KF289074
*P.brazilianiae*	LGMF 330 ^T^	*Mangiferaindica*	Brazil	JF343572	KF206217	JF343593	JF343656	JF343758
	LGMF 334	*Mangiferaindica*	Brazil	JF343566	KF206215	JF343587	JF343650	JF343752
*P.capitalensis*	CBS 114751	*Vaccinium* sp.	New Zealand	EU167584	EU167584	FJ538407	FJ538465	KF289088
	CBS 128856 ^T^	*Stanhopea* sp.	Brazil	JF261465	KF206304	JF261507	JF343647	JF343776
*P.carochlae*	CGMCC 3.17317 ^T^	*Caryotaochlandra*	China	KJ847422	NA	KF289178	KF289273	KF289092
*P.cavendishii*	BRIP 57384	*Musa* cv. *Lady finger*	Australia	KC117644	KU697330	KF009695	KF014059	KU716085
	BRIP 57383	*Musa* cv. *Lady finger*	Australia	KC117643	KU697329	KF009694	KF014058	KU716084
*P.cordylinophila*	MFLUCC 10-0166 ^T^	*Cordylinefruticosa*	Thailand	KF170287	KF206242	KF289172	KF289295	KF289076
	MFLUCC 12-0014	*Cordylinefruticosa*	Thailand	KF170288	KF206228	KF289171	KF289301	KF289075
*P.doitungensis*	MFLU 21-0175 ^T^	*Dasymaschalonobtusipetalum*	Thailand	OK661033	OK661034	OL345581	NA	NA
*P.eugeniae*	CBS 445.82 ^T^	*Eugeniaaromatica*	Indonesia	AY042926	KF206288	KF289208	KF289246	KF289139
*P.fallopiae*	MUCC0113 ^T^	*Fallopiajaponica*	Japan	AB454307	NA	NA	AB704228	NA
*P.guangdongensis*	CFCC 58144 ^T^	*Viburnumodoratissimum*	China	OQ202160	OQ202170	OQ267758	OQ267764	OQ267770
	CFCC 58766	*Viburnumodoratissimum*	China	OQ202161	OQ202171	OQ267759	OQ267765	OQ267771
	CFCC 58772	*Viburnumodoratissimum*	China	OQ202162	OQ202172	OQ267760	OQ267766	OQ267772
*P.ilicis-aquifolii*	CGMCC 3.14358 ^T^	*Ilexaquifolium*	China	JN692538	NA	JN692526	JN692514	NA
	CGMCC 3.14359	*Ilexaquifolium*	China	JN692539	NA	JN692527	JN692515	NA
*P.maculata*	CPC 18347 ^T^	*Musa* cv. *Golygoly pot-pot*	Australia	JQ743570	NA	KF009700	KF014016	NA
	BRIP 46622	*Musa* cv. *Golygoly pot-pot*	Australia	JQ743567	NA	KF009692	KF014013	NA
*P.mangiferae*	IMI 260576 ^T^	*Mangiferaindica*	India	JF261459	KF206222	JF261501	JF343641	JF343748
*P.mangifera-indicae*	MFLUCC 10-0029 ^T^	*Mangiferaindica*	Thailand	KF170305	KF206240	KF289190	KF289296	KF289121
*P.musaechinensis*	GZAAS 6.1247	*Musa* sp.	China	KF955294	NA	KM816639	KM816627	KM816633
	GZAAS 6.1384	*Musa* sp.	China	KF955295	NA	KM816640	KM816628	KM816634
*P.musarum*	BRIP 57803	*Musa* sp.	Malaysia	JX997138	NA	KF009737	KF014055	NA
	BRIP 58028	*Musa* sp.	Australia	KC988377	NA	KF009738	KF014054	NA
*P.oblongifoliae*	SAUCC210055	*Garciniaoblongifolia*	China	OM248442	OM232085	OM273890	OM273894	OM273898
	SAUCC210052 ^T^	*Garciniaoblongifolia*	China	OM248445	OM232088	OM273893	OM273897	OM273901
*P.paracapitalensis*	CPC 26517 ^T^	*Citrusfloridana*	Italy	KY855622	KY855796	KY855951	KY855677	KY855735
	CPC 26518	*Citrusfloridana*	Italy	KY855623	KY855797	KY855952	KY855678	KY855736
*P.parthenocissi*	CBS 111645 ^T^	*Parthenocissusquinquefolia*	USA	EU683672	NA	JN692530	JN692518	NA
*P.partricuspidatae*	NBRC 9466 ^T^	*Parthenocissustricuspidata*	Japan	KJ847424	NA	KJ847446	KJ847432	KJ847440
	NBRC 9757	*Parthenocissustricuspidata*	Japan	KJ847425	NA	KJ847447	KJ847433	KJ847441
*P.philoprina*	CBS 587.69	*Ilexaquifolium*	Spain	KF154278	KF206297	KF289206	KF289250	KF289137
*P.phoenicis*	CBS 147091	*Phoenixreclinata*	South Africa	MW883442	MW883833	MW890098	MW890031	MW890050
*P.pterospermi*	SAUCC210104 ^T^	*Pterospermumheterophyllum*	China	OM249954	OM249956	OM273902	OM273904	OM273906
	SAUCC210106	*Pterospermumheterophyllum*	China	OM249955	OM249957	OM273903	OM273905	OM273907
*P.rhizophorae*	NCYUCC 19-0352 ^T^	*Rhizophorastylosa*	China	MT360030	MT360039	NA	MT363248	MT363250
	NCYUCC 19-0358	*Rhizophorastylosa*	China	MT360031	MT360040	NA	MT363249	MT363251
*P.schimae*	CGMCC 3.14354 ^T^	*Schimasuperba*	China	JN692534	NA	JN692522	JN692510	JN692506
*P.schimicola*	CGMCC 3.17319 ^T^	*Schimasuperba*	China	KJ847426	NA	KJ847448	KJ847434	KJ854895
	CGMCC 3.17320	*Schimasuperba*	China	KJ847427	NA	KJ847449	KJ847435	KJ854896
*P.styracicola*	CGMCC3.14985 ^T^	*Styraxgrandiflorus*	China	JX025040	NA	JX025045	JX025035	JX025030
	CGMCC3.14989	*Styraxgrandiflorus*	China	JX025041	NA	JX025046	JX025036	JX025031
*P.vitis-rotundifoliae*	CGMCC 3.17322 ^T^	*Vitisrotundifolia*	USA	KJ847428	NA	KJ847450	KJ847436	KJ847442
	CGMCC 3.17321	*Vitisrotundifolia*	USA	KJ847429	NA	KJ847451	KJ847437	KJ847443
***Phyllostictaconcentrica* species complex**								
*P.anhuiensis*	CFCC 54840^T^	*Quercusaliena*	China	OQ202157	OQ202167	OQ267761	OQ267767	OQ267773
	CFCC 55887	*Quercusaliena*	China	OQ202158	OQ202168	OQ267762	OQ267768	OQ267774
	CFCC 58849	*Quercusaliena*	China	OQ202159	OQ202169	OQ267763	OQ267769	OQ267775
*P.aspidistricola*	NBRC 102244 ^T^	*Aspidistraelatior*	Japan	AB454314	NA	NA	AB704204	NA
*P.aucubae-japonicae*	MAFF 236703 ^T^	*Aucubajaponica*	Japan	KR233300	NA	KR233310	KR233305	NA
*P.bifrenariae*	CBS 128855 ^T^	*Bifrenariaharrisoniae*	Brazil	JF343565	KF206209	JF343586	JF343649	JF343744
	CPC 17467	*Bifrenariaharrisoniae*	Brazil	KF170299	KF206260	KF289207	KF289283	KF289138
*P.catimbauensis*	URM 7672 ^T^	*Mandevillacatimbauensis*	Brazil	MF466160	MF466163	MF466155	MF466157	NA
	URM 7674	*Mandevillacatimbauensis*	Brazil	MF466161	MF466164	MF466153	MF466158	NA
*P.citriasiana*	CBS 120486 ^T^	*Citrusmaxima*	Thailand	FJ538360	KF206314	FJ538418	FJ538476	JF343686
*P.citriasiana*	CBS 120487	*Citrusmaxima*	China	FJ538361	KF206313	FJ538419	FJ538477	JF343687
*P.citribraziliensis*	CBS 100098 ^T^	*Citruslimon*	Brazil	FJ538352	KF206221	FJ538410	FJ538468	JF343691
*P.citricarpa*	CBS 127454 ^T^	*Citruslimon*	Australia	JF343583	KF206306	JF343604	JF343667	JF343771
*P.citrichinensis*	ZJUCC 200956 ^T^	*Citrusreticulata*	China	JN791620	NA	JN791459	JN791533	NA
	ZJUCC 2010150	*Citrusmaxima*	China	JN791662	NA	JN791514	JN791582	NA
*P.citrimaxima*	MFLUCC 10-0137 ^T^	*Citrusmaxima*	Thailand	KF170304	KF206229	KF289222	KF289300	KF289157
*P.concentrica*	CBS 937.70	*Hederahelix*	Italy	FJ538350	KF206291	FJ538408	KF289257	JF411745
	CPC 18842 ^T^	*Hedera* sp.	Italy	KF170310	KF206256	KF289228	KF289288	KF289163
*P.cussonia*	CPC 14873 ^T^	*Cussonia* sp.	South Africa	JF343578	KF206279	JF343599	JF343662	JF343764
	CPC 14875	*Cussonia* sp.	South Africa	JF343579	KF206278	JF343600	JF343663	JF343765
*P.elongata*	CBS 126.22 ^T^	*Oxycoccusmacrocarpos*	USA	FJ538353	NA	FJ538411	FJ538469	KF289164
*P.ericarum*	CBS 132534 ^T^	*Ericagracilis*	South Africa	KF206170	KF206253	KF289227	KF289291	KF289162
*P.gardeniicola*	MUCC0117	*Gardeniajasminoides*	Japan	AB454310	NA	NA	AB704230	NA
	MUCC0089	*Gardeniajasminoides*	Japan	AB454303	NA	NA	NA	NA
*P.gwangjuensis*	CNUFC NJ1-12 ^T^	*Torreyanucifera*	Korea	OK285195	NA	OM038511	OM001471	NA
	CNUFC NJ1-12-1	*Torreyanucifera*	Korea	OK285196	NA	OM038512	OM001472	NA
*P.hostae*	CGMCC 3.14355 ^T^	*Hostaplantaginea*	China	JN692535	NA	JN692523	JN692511	JN692503
	CGMCC 3.14356	*Hostaplantaginea*	China	JN692536	NA	JN692524	JN692512	JN692504
*P.hymenocallidicola*	CBS 131309 T	*Hymenocallislittoralis*	Australia	JQ044423	JQ044443	KF289211	KF289242	KF289142
	CPC 19331	*Hymenocallislittoralis*	Australia	KF170303	KF206254	KF289212	KF289290	KF289143
*P.hypoglossi*	CBS 101.72	*Ruscusaculeatus*	Italy	FJ538365	KF206326	FJ538423	FJ538481	JF343694
	CBS 434.92 ^T^	*Ruscusaculeatus*	Italy	FJ538367	KF206299	FJ538425	FJ538483	JF343695
*P.iridigena*	CBS 143410 ^T^	*Iris* sp.	South Africa	MG934459	NA	MG934502	MG934466	NA
*P.kerriae*	MAFF 240047 ^T^	*Kerriajaponica*	Japan	AB454266	NA	NA	NA	NA
*P.kobus*	MUCC0049	*Magnoliakobus*	Japan	AB454286	NA	NA	AB704221	NA
*P.ophiopogonis*	KACC 47754	*Ophiopogonjaponicus*	South Korea	KP197057	NA	NA	NA	NA
	LrLF11	*Lycorisradiata*	China	MG543713	NA	NA	NA	NA
*P.paracitricarpa*	CPC 27169 ^T^	*Citruslimon*	Greece	KY855635	KY855809	KY855964	KY855690	KY855748
	ZJUCC 200933	*Citrussinensis*	China	JN791626	KY855813	JN791468	JN791544	KY855752
*P.pilospora*	MUCC 2912a ^T^	Chamaecyparispisiferavar.plumose	Japan	LC542597	LC543423	LC543445	LC543465	NA
*P.speewahensis*	BRIP 58044 ^T^	Orchids	Australia	KF017269	NA	KF017268	NA	NA
*P.spinarum*	CBS 292.90	*Chamaecyparispisifera*	France	JF343585	KF206301	JF343606	JF343669	JF343773
*P.westeae*	BRIP 72390c ^T^	*Clerodendruminerme*	Australia	OP599631	NA	OP627090	NA	NA
***Phyllostictacruenta* species complex**								
*P.abieticola*	CBS 112067	*Abiesconcolor*	Canada	KF170306	EU754193	NA	KF289238	NA
*P.cornicola*	CBS 111639	*Cornusflorida*	USA	KF170307	NA	NA	KF289234	NA
*P.cruenta*	CBS 858.71	*Polygonatumodoratum*	Czech Republic	MG934458	NA	MG934501	MG934465	MG934474
*P.cruenta*	MUCC0206	Polygonatumodoratumvar.pluriflorum	Japan	AB454331	NA	NA	AB704237	NA
*P.cryptomeriae*	KACC 48643	Juniperuschinensisvar.sargentii	Not given	MK396559	NA	NA	NA	NA
	MUCC0028	*Cryptomeriajaponica*	Japan	AB454271	NA	NA	AB704213	NA
*P.foliorum*	CBS 447.68 ^T^	*Taxusbaccata*	Netherlands	KF170309	KF206287	KF289201	KF289247	KF289132
*P.gaultheriae*	CBS 447.70 ^T^	*Gaultheriahumifusa*	USA	JN692543	KF206298	JN692531	KF289248	JN692508
*P.hakeicola*	CBS 143492 ^T^	*Hakea* sp.	Australia	MH107907	MH107953	MH108025	MH107984	MH107999
*P.hamamelidis*	MUCC149	*Hamamelisjaponica*	Japan	KF170289	NA	NA	KF289309	NA
*P.hubeiensis*	CGMCC 3.14986 ^T^	*Viburnumodoratissimim*	China	JX025037	NA	JX025042	JX025032	JX025027
	CGMCC 3.14987	*Viburnumodoratissimim*	China	JX025038	NA	JX025043	JX025033	JX025028
*P.illicii*	24-1-1 T	*Illiciumverum*	China	MF198235	MF198240	MF198237	MF198243	NA
	16-16-1	*Illiciumverum*	China	MF198234	MF198239	MF198236	MF198242	NA
*P.leucothoicola*	MUCC553 ^T^	*Leucothoecatesbaei*	Japan	AB454370	AB454370	NA	KF289310	NA
*P.ligustricola*	MUCC0024 ^T^	*Ligustrumobtusifolium*	Japan	AB454269	NA	NA	AB704212	NA
*P.minima*	CBS 585.84 ^T^	*Acerrubrum*	USA	KF206176	KF206286	KF289204	KF289249	KF289135
*P.neopyrolae*	CPC 21879 ^T^	*Pyrolaasarifolia*	Japan	AB454318	AB454318	NA	AB704233	NA
*P.pachysandricola*	MUCC124 ^T^	*Pachysandraterminalis*	Japan	AB454317	AB454317	NA	AB704232	NA
*P.paxistimae*	CBS 112527 ^T^	*Paxistimamysinites*	USA	KF206172	KF206320	KF289209	KF289239	KF289140
*P.podocarpicola*	CBS 728.79 ^T^	*Podocarpusmaki*	USA	KF206173	KF206295	KF289203	KF289252	KF289134
*P.pyrolae*	IFO 32652	*Ericacarnea*	Not given	AB041242	NA	NA	NA	NA
*P.rubella*	CBS 111635 ^T^	*Acerrubrum*	USA	KF206171	EU754194	KF289198	KF289233	KF289129
*P.sphaeropsoidea*	CBS 756.70	*Aesculushippocastanum*	Germany	AY042934	KF206294	KF289202	KF289253	KF289133
*P.telopeae*	CBS 777.97 ^T^	*Telopeaspeciosissima*	Tasmania	KF206205	KF206285	KF289210	KF289255	KF289141
*P.yuccae*	CBS 112065	*Yuccaelephantipes*	USA	KF206175	NA	NA	KF289237	NA
	CBS 117136	*Yuccaelephantipes*	New Zealand	JN692541	KF766385	JN692529	JN692517	JN692507
***Phyllostictaowaniana* species complex**								
*P.austroafricana*	CBS 144593 ^T^	leaf spots of unidentified deciduous tree	South Africa	MK442613	MK442549	MK442704	MK442640	NA
*P.carissicola*	CPC 25665 ^T^	*Carissamacrocarpa*	South Africa	KT950849	KT950863	KT950879	KT950872	KT950876
*P.hagahagaensis*	CBS 144592 ^T^	*Carissabispinosa*	South Africa	MK442614	MK442550	MK442705	MK442641	MK442657
*P.owaniana*	CBS 776.97 ^T^	*Brabejumstellatifolium*	South Africa	FJ538368	KF206293	FJ538426	KF289254	JF343767
	CPC 14901	*Brabejumstellatifolium*	South Africa	JF261462	KF206303	JF261504	KF289243	JF343766
*P.podocarpi*	CBS 111646	*Podocarpusfalcatus*	South Africa	AF312013	KF206323	KC357671	KC357670	KF289169
	CBS 111647	*Podocarpuslanceolata*	South Africa	KF154276	KF206322	KF289232	KF289235	KF289168
*P.pseudotsugae*	CBS 111649	*Pseudotsugamenziesii*	USA	KF154277	KF206321	KF289231	KF289236	KF289167
***Phyllostictarhodorae* species complex**								
*P.mimusopisicola*	CBS 138899 ^T^	*Mimusopszeyheri*	South Africa	KP004447	MH878626	NA	NA	NA
*P.rhodorae*	CBS 901.69	*Rhododendron* sp.	Netherlands	KF206174	KF206292	KF289230	KF289256	KF289166
***Phyllostictavaccinii* species complex**								
*P.vaccinii*	ATCC 46255 ^T^	*Vacciniummacrocarpon*	China	KC193585	NA	KC193582	KC193580	KC193583
	LC 2795	*Vitismacrocarpon*	USA	KR233323	NA	NA	NA	NA
*P.vacciniicola*	CPC 18590 ^T^	*Vacciniummacrocarpum*	USA	KF170312	KF206257	KF289229	KF289287	KF289165
**Outgroup**								
*B.obtusa*	CMW 8232 ^T^	Conifers	South Africa	AY972105	NA	DQ280419	AY972111	NA
*B.stevensii*	CBS 112553 ^T^	culture from isotype of *Diplodiamutila*	Not given	AY259093	AY928049	AY573219	NA	NA

Notes: *^T^ = ex-type strains, NA = not available.

## ﻿Results

### ﻿Phylogenetic analyses

**Figure 1.**
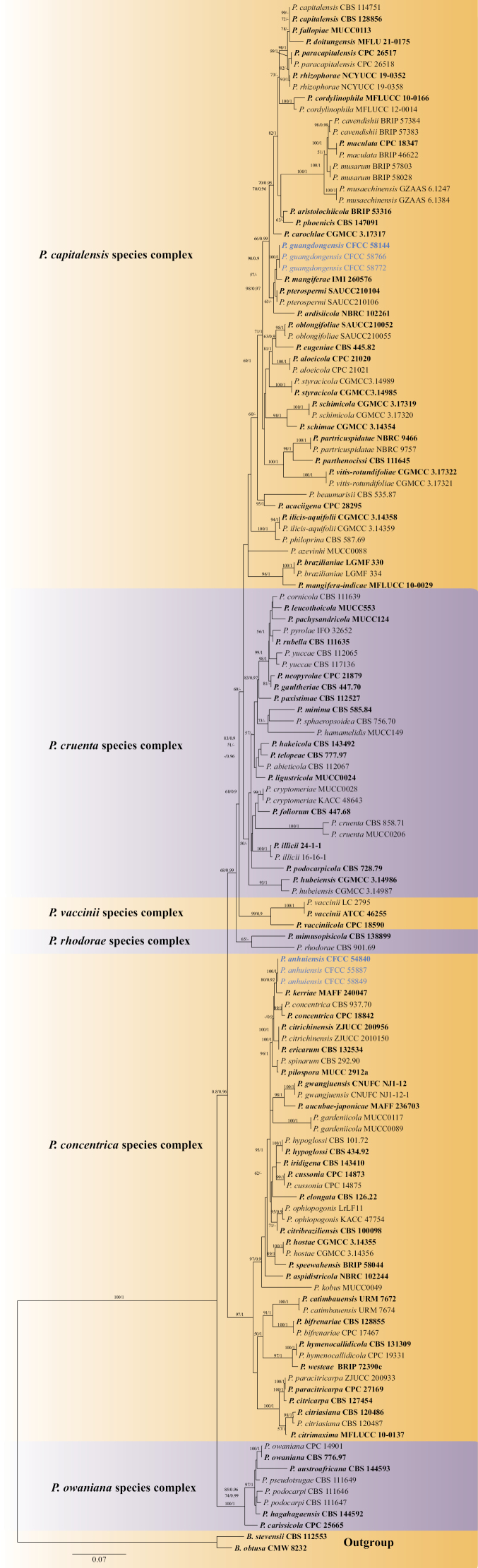
Phylogram of *Phyllosticta* genus resulting from a maximum likelihood analysis based on a combined matrix of ITS, LSU, *tef1*, *act* and *gapdh* loci. The tree is artificially rooted to *B.obtusa* (CMW 8232) and *B.stevensii* (CBS 112553). ML bootstrap values (left, ML-BS ≥ 50%) and Bayesian posterior probabilities (right, BYPP ≥ 0.9) are given at the nodes. Ex-type strains are indicated in bold. Strains from the present study are marked in blue.

In this study, phylogenetic analyses contained sequences from 131 fungal samples representing 93 taxa, including two outgroup taxa, viz., *Botryosphaeriaobtusa* (CMW 8232) and *B.stevensii* (CBS 112553). The multi-locus datasets comprised 2460 characters including gaps, 521 for ITS, 764 for LSU, 297 for *tef1*, 248 for *act* and 630 for *gapdh*, with 1499/2460 conserved sites, 187/2460 variable sites, and 774/2460 parsimony informative. The best scoring RAxML tree with a final likelihood value of -22751.44. Estimated base frequencies were: A = 0.206387, C = 0.294301, G = 0.279093, T = 0.220219; substitution rates AC = 1.049607, AG = 3.135926, AT = 1.344881, CG = 1.068545, CT = 6.294467, GT = 1.00000; gamma distribution shape parameter α = 0.690585. In the phylogenetic tree (Fig. 1), *Phyllosticta* was divided into six distinct lineages as six species complexes, and our isolates formed two separate lineages represented two new species viz., *P.anhuiensis* (CFCC 54840, CFCC 55887 and CFCC 58849) and *P.guangdongensis* (CFCC 58144, CFCC 58766 and CFCC 58772).

### ﻿Taxonomy

#### ﻿ Phyllosticta anhuiensis

Taxon classificationFungiBotryosphaerialesPhyllostictaceae

Ning Jiang & C.B. Wang sp. nov.

81C08238-FAAB-5DCF-83D9-64BD9462A468

 847160

Fig. 2

##### Etymology.

Referring to the Anhui Province, where the species was first collected.

##### Description.

***Sexual morph***: Unknown. ***Asexual morph***: Conidiomata pycnidial, aggregated, black, erumpent, globose to pyriform, exuding gray to pale yellow conidial masses, 100–400 µm diam. Conidiophores subcylindrical to ampulliform, reduced to conidiogenous cells. Conidiogenous cells phialidic, hyaline, thin-walled, smooth, subcylindrical to ampulliform, 10–16 × 2.5–4.5 μm. Conidia 8.5–12 × 5.5–9 μm, (mean ± SD = 10 ± 1 × 7.2 ± 0.7 μm), solitary, hyaline, aseptate, thin and smooth-walled, coarsely guttulate, globose or ellipsoid to obvoid, enclosed in a thin persistent sheath, 1–1.5 μm thick, and bearing an apical mucoid appendage 4–6 × 1–2 μm, flexible, unbranched, tapering towards an acutely rounded tip.

**Figure 2.**
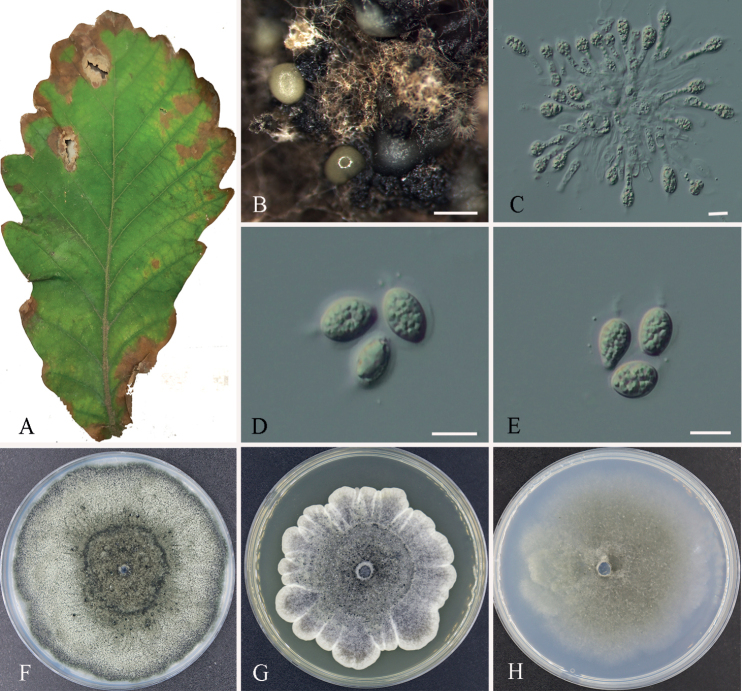
Morphology of *Phyllostictaanhuiensis* (CFCC 54840) **A** diseased leaf of *Quercusaliena***B** conidiomata **C** conidiogenous cells **D, E** conidia **F–H** colonies on PDA, MEA and SNA after two weeks at 25 °C. Scale bars: 500 μm (**B**); 10 μm (**C–E**).

##### Culture characters.

Colonies on PDA flat, with irregular edge, slow growing, grayish-green to green, reaching a 90 mm diameter after two weeks. Colonies on MEA flat, undulate at the edge, slow growing, gray-white to gray, reaching a 70–80 mm diameter after two weeks. Colonies on SNA flat, slow growing, celandine green, reaching a 60–70 mm diameter after two weeks.

##### Specimens examined.

China, Anhui Province, Hefei City, leaf spots of *Quercusaliena*, Yong Li & Dan-ran Bian, 10 August 2019 (holotype CAF800072; ex-type culture: CFCC 54840). Ibid. (cultures: CFCC 55887 and CFCC 58849).

##### Notes.

In the phylogeny analyses, *P.anhuiensis* groups sister to *P.kerriae* (MAFF 240047). *P.kerriae* was associated with *Kerriajaponica* in Japan (Motohashi et al. 2008). Comparison of DNA sequences of *P.anhuiensis* with *P.kerriae* (MAFF 240047), there is 99.4% (447/480 identities; 0/480 gaps) sequence similarity in ITS, 99.8% (554/555 identities, 0/480 gaps) in LSU, 98.6% (215/218 identities, 0/218 gaps) in *tef1*, and 97.7% (212/217 identities, 0/217 gaps) in *act*. Morphologically, *P.anhuiensis* can be distinguished from *P.kerriae* in having shorter appendage (4–6 µm in *P.anhuiensis* vs. 5–12.5 µm in *P.kerriae*) (Motohashi et al. 2008). Therefore, this species was regarded as a new species based on morphology and multi-locus phylogeny.

#### ﻿ Phyllosticta guangdongensis

Taxon classificationFungiBotryosphaerialesPhyllostictaceae

Ning Jiang & C.B. Wang sp. nov.

A5308D23-E690-5AFA-B6FD-EE50F3BABB27

 847161

Fig. 3

##### Etymology.

Referring to the Guangdong Province, where the species was first collected.

##### Description.

***Sexual morph***: Unknown. ***Asexual morph***: Conidiomata pycnidial, aggregated, black, globose to pyriform, exuding opaque conidial masses, erumpent, 100–450 µm diam. Conidiophores subcylindrical to ampulliform, reduced to conidiogenous cells. Conidiogenous cells phialidic, subcylindrical to ampulliform, hyaline, smooth, 10–15 × 2.5–4 μm. Conidia 10–14 × 6–8 μm, (mean ± SD = 11.5 ± 1.3 × 7.5 ± 0.6 μm), solitary, hyaline, aseptate, thin and smooth-walled, ellipsoid to obovoid, coarsely guttulate, enclosed in a thin persistent mud sheath, 1–1.5 μm thick, with an apical mucoid appendage, 4.5–10 × 1–2 μm, flexible, unbranched, tapering towards an acutely rounded tip.

**Figure 3.**
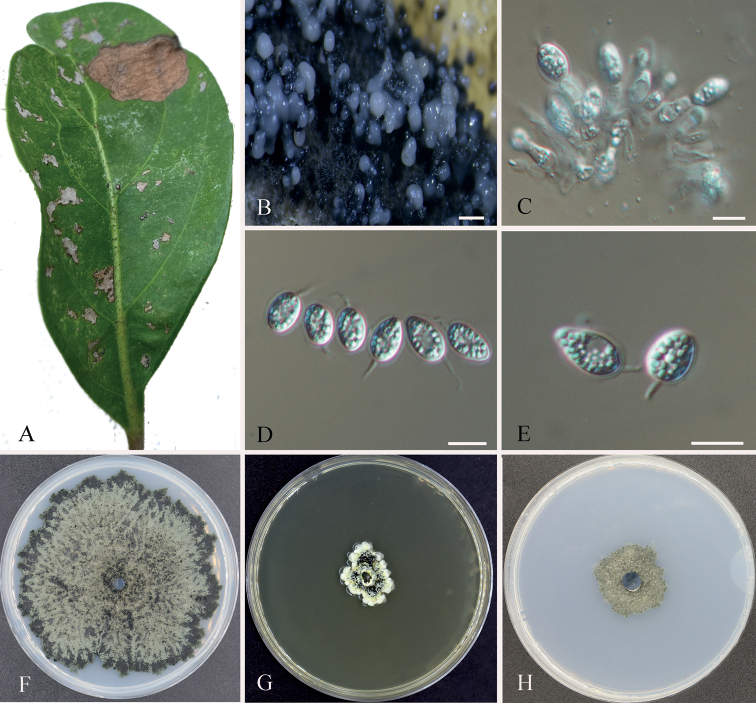
Morphology of *Phyllostictaguangdongensis* (CFCC 58144) **A** diseased leaf of *Viburnumodoratissimum***B** conidiomata **C** conidiogenous cells **D, E** conidia **F–H** colonies on PDA, MEA and SNA after two weeks at 25 °C. Scale bars: 500 μm (**B**); 10 μm (**C–E**).

##### Culture characters.

Colonies on PDA flat, slow growing, grayish-green in the center, and dark green at margin reaching 85 mm diameter after two weeks. Colonies on MEA slow growing, yellow in the center, white at undulate the margin, reaching a 20–25 mm diameter after two weeks. Colonies on SNA flat, slow growing, grayish-green, reaching a 25–30 mm diameter after two weeks.

##### Specimens examined.

China, Guangdong Province, Guangzhou City, leaf spot of *Viburnumodoratissimum*, Yong Li, 20 September 2022 (holotype CAF800073; ex-type culture: CFCC 58144). Ibid. (cultures: CFCC 58766 and CFCC 58772).

##### Notes.

Phylogeny indicates that *P.anhuiensis* groups sister to *P.mangiferae* (IMA 260576). *P.mangiferae* was associated with *Mangiferaindica* leaves in Tanzania (Ebbels and Allen 1979; Glienke et al. 2011). Comparison of DNA sequences of *P.anhuiensis* with *P.mangiferae* (IMA 260576), there are 99.1% (471/475 identifies, 0/475 gaps) sequence similarity in ITS, 99.6% (760/763 identifies, 0/763 gaps) in LSU, 97.7% (211/216 identifies, 2/218 gaps) in *tef1*, 98.2% (221/225 identifies, 0/225 gaps) in *act*, and 98.4% (614/624 identifies, 6/624 gaps) in *gapdh*. Morphologically, *P.guangdongensis* can be distinguished from *P.mangiferae* in longer conidia (10–14 μm in *P.guangdongensis* vs. 8–12 µm in *P.mangiferae*) and shorter appendage (4.5–10 µm in *P.guangdongensis* vs. 7–13 µm in *P.mangiferae*) (Glienke et al. 2011). Therefore, this species was regarded as a new species based on morphology and multi-locus phylogenetic analyses.

## ﻿Discussion

*Phyllosticta* is a species-rich genus with more than 3211 records listed in the Index Fungorum (http://www.indexfungorum.org). For the *Phyllosticta* species identification, molecular data have proven useful in resolving species relationships (Okane et al. 2003; Su and Cai 2012; Guarnaccia et al. 2017; Norphanphoun et al. 2020; Zhang et al. 2022). ITS is a genetic marker for genus level, and combining it with additional loci (LSU, *tef1*, *act* and *gapdh*) is enough for species-level resolution (Jayawardena et al. 2019; Norphanphoun et al. 2020). In this study, based on the phylogenetic analyses of presently accepted species using five loci (ITS, LSU, *tef1*, *act* and *gapdh*), there are six species complexes and 93 species accepted in *Phyllosticta* (Table 1), viz., *P.capitalensis* species complex (including 33 species), *P.concentrica* species complex (including 28 species), *P.cruenta* species complex (including 22 species), *P.owaniana* species complex (including six species), *P.rhodorae* species complex (including two species), and *P.vaccinii* species complex species complex (including two species). *P.anhuiensis* and *P.guangdongensis* formed two well separated clades in the *P.concentrica* and *P.capitalensis* species complexes, distinguishing from all accepted species in this genus by DNA sequences data.

Morphologically, our isolates have the typical structure of *Phyllosticta* (van der Aa and Vanev 2002). The asexual morph of species in the *P.concentrica* species complex is characterized by globose or ellipsoid to obvoid conidia enclosed in a thin persistent sheath with an apical mucoid appendage (Norphanphoun et al. 2020). The asexual morph of species in the *P.capitalensis* species complex are characterized by ellipsoid or ellipsoid to obovoid, ovoid, obpyriform conidia with a mucoid sheath with an apical mucoid appendage (Norphanphoun et al. 2020). Our isolates include the essential characteristics of their species complexes, and differ from their closest relatives by the size ranges of conidia and appendage (Motohashi et al. 2008; Glienke et al. 2011).

*Phyllostictaanhuiensis* was isolated from *Q.aliena* in Anhui Province, and *P.guangdongensis* was isolated from *V.odoratissimum* in Guangdong Province. Among *Phyllosticta* species recorded from *Quercus* and *Viburnum* with sequence date and morphological features, *P.capitalensis* was isolated from *Q.dentata* and *Q.variabilis* in Japan; *P.concentrica* was isolated from *Q.robur* in Poland and *Q.ilex* in Ukraine; and *P.hubeiensis* was isolated from *V.odoratissimum* in China (Okane et al. 2003; Mulenko et al. 2008; Zhang et al. 2013; Farr and Rossman 2022). *P.capitalensis* and *P.concentrica* are common species reported from various plants, and *P.hubeiensis* was only recorded from *V.odoratissimum* (Wikee et al. 2013a, b; Zhang et al. 2013; Farr and Rossman 2022). Our isolates formed individual lineages as shown in Fig. 1, segregated from those three species. Morphologically, *P.anhuiensis* differs from *P.capitalensis* and *P.concentrica* by having longer conidiogenous cells (10–16 × 2.5–4.5 μm in *P.anhuiensis* vs. 7–10 × 3–5 in *P.capitalensis* vs. 7–10 × 3–6 μm in *P.concentrica*), shorter conidia (8.5–12 × 5.5–9 μm in *P.anhuiensis* vs. 10–14 × 5–7 μm in *P.capitalensis* vs. 10–14 × 6–9 μm in *P.concentrica*) and shorter appendage (4–6 × 1–2 μm in *P.anhuiensis* vs. 5–15 × 1–1.5 μm in *P.concentrica*) (Glienke et al. 2011; Wikee et al. 2013a); *P.guangdongensis* can be distinguished from *P.hubeiensis* in having shorter appendage (4.5–10 µm in *P.guangdongensis* vs. 7–12 µm in *P.hubeiensis*) (Zhang et al. 2013).

In this study, we introduced two novel species from forestry trees. Previously, many *Phyllosticta* species were found in economic hosts, and with the investigation and study of *Phyllosticta*, many *Phyllosticta* will be found on forestry trees and this will improve our understanding of the species diversity.

## Supplementary Material

XML Treatment for Phyllosticta anhuiensis

XML Treatment for Phyllosticta guangdongensis
